# Effect of size and charge asymmetry on aggregation kinetics of oppositely charged nanoparticles

**DOI:** 10.1038/s41598-019-40379-y

**Published:** 2019-03-06

**Authors:** Kulveer Singh, Anubhav Raghav, Prateek K. Jha, Soumitra Satapathi

**Affiliations:** 1 Department of Physics, IIT Roorkee, Roorkee, Uttarakhand 246777 India; 2 Centre of Nanotechnology, IIT Roorkee, Roorkee, Uttarakhand 247667 India; 3 Department of Chemical Engineering, IIT Roorkee, Roorkee, 247667 Uttarakhand India

## Abstract

We report a theoretical and experimental study of the aggregation kinetics of oppositely charged nanoparticles. Kinetic Monte Carlo simulations are performed for symmetric, charge-asymmetric and size-asymmetric systems of oppositely charged nanoparticles. Simulation results show that both the weight and number average aggregate size kinetics exhibit power law scaling with different exponents for small and intermediate time of evolution. The qualitative behavior of the symmetric and the size asymmetric system are the same, but the charge asymmetric system shows anomalous behavior for intermediate to high particle concentrations. We also observe a strong dependence of power law exponents on the particle concentration. Radius of gyration of the cluster that indicates how nanoparticles inside a cluster are distributed around the center of mass of the cluster shows a non-monotonic time evolution with pronounced peak at higher particle concentration. The dependence of particle concentration on aggregation kinetics as observed by predictive numerical simulation is further verified experimentally by monitoring the time evolution of aggregate size of nanoparticles assemblies of Poly (methacrylic acid) (PMMA) nanoparticles functionalized with oppositely charged ligands. These size and charge tunable asymmetric polymeric nanoparticles were synthesized by modified miniemulsion technique. The integrated approach for studying nanoparticles aggregation as described here renders new insights into super structure formation and morphology optimization which can be potentially useful in the design of new materials, such as organic photovoltaics.

## Introduction

Bottom-up self-assembly to build functional complex structures using nanoparticles (NP) as building blocks has received tremendous research attention in last few years^1^. These self-assembled structures can be effectively used in various industrial applications such as waste water treatment^2^, sensing^3^, drug delivery^4^, NP based solar cells^5^, etc. Tuning of NP shape, size, interaction etc. gives a control over the super structure formed by them^6–9^. Aggregation of NPs is strongly affected by the competition of inter-particle interactions such as van der Waals, electrostatic, and magnetic interactions, which in turn depends on the material property of NPs, dielectric constant of solvent, temperature, pH, and presence of external fields. Out of various inter-particle interactions, electrostatic interactions are somewhat unique, because they are long-ranged and, can be either attractive or repulsive. Also, their interaction strength and interaction range can be easily tuned by varying the charge on the NPs and ionic strength, respectively^9–13^. Such tunability of electrostatic interaction has many implications on the aggregation kinetics and self-assembly of oppositely charged NPs^14^.

Aggregation of nanoparticles can be broadly classified into homo-aggregation and hetero-aggregation^15^; homo-aggregation is the aggregation of the NPs with identical characteristics, whereas in hetero-aggregation, NPs with different physical and chemical properties aggregates to form clusters. Naturally, hetero-aggregation is more complex and is therefore not yet completely understood^15–18^. Recent experiments on the synthesis of NPs of different size, charge, and chemical composition have provided new insight into the mechanisms of aggregation processes^19–21^. Experimentally aggregation kinetics of NPs is studied using Dynamic Light Scattering(DLS)^22,23^, time-resolved DLS^24^ and Small-angle Neutron Scattering(SANS)^25,26^. Charged NPs hetero-aggregation has received much more attention due to ease of controlling the charge on NPs and its application in ionic colloidal crystals, organic optoelectronics and flocculation techniques^27–32^. However, synthesis of monodisperse and precisely charged NPs at large scale with high reproducibility is always challenging and needs in-depth theoretical understanding and predictive modeling. Several theoretical models^22,23,29,33^ to explain aggregation of sub-micrometer spherical particles have been developed and experimentally validated, the most common being DLVO theory. Computer simulation plays a crucial role to understand the internal dynamics of the system and provide a complimentary tool for the rational design of chemical reactors for high-throughput synthesis of nanoparticles.

Monte Carlo(MC) simulation is very useful tool to study the final equilibrium structure of super lattice formed by aggregation of NPs and the effect of various interactions on these structures^12^. Using importance sampling of configuration space, MC simulation efficiently reaches the final equilibrium state by skipping several metastable states. But during the evolution to reach equilibrium, system passes through various non-equilibrium complex structures which are very different from equilibrium structures^34^. For instance, various studies have shown that system of oppositely charged nanoparticles are often trapped in non-equilibrium “gel”-like (percolated) structures at high packing fractions, and do not evolve to minimum energy crystalline structures due to slow system dynamics^35–37^. In one of our previous study, we have found that these kinetically trapped percolated structures formed by self-assembly of electron conducting and hole conducting polymer NPs are the bulk heterojunction morphologies for efficient organic solar cells^37^.

In this paper, using kinetic Monte Carlo (kMC) simulations, we study the effect of charge and size asymmetry on aggregation kinetics of oppositely charged NPs. kMC scheme used in this simulation has been used in various other studies^36–39^ and is argued to be more efficient than Brownian dynamics simulation because it employs a longer time step without compromising the numerical stability. The predictive modeling is validated experimentally by synthesizing size and charge tunable functionalized polymeric NPs with modified mini-emulsion method. Polymeric NPs functionalized with oppositely charged surfactants are chosen as model system to study aggregation behavior as they can be easily synthesized with different sizes and charges by tuning polymer’s molecular weight, polymer to surfactant ratio, sonication time, solvent evaporation rate etc. The time evolution of the aggregate size of these PMMA NPs is studied by DLS.

## Methodology

### Numerical Simulation (Model and Method)

Implicit-solvent simulations are performed on the solution containing two types, type A (+) and type B (−), of oppositely charged spherical NPs of diameter *σ*_*A*_ and *σ*_*B*_ and valence *z*_*A*_ and *z*_*B*_, respectively. The system containing total *N*_*T*_ NPs satisfy the electroneutrality condition *n*_*A*_*z*_*A*_ + *n*_*B*_*z*_*B*_ = 0 and *n*_*A*_ + *n*_*B*_ = *N*_*T*_, where *n*_*A*_ and *n*_*B*_ are number of NPs of type A and type B respectively. NPs interact via electrostatic interactions, which are modeled using a pairwise screened Coulomb potential between *i*^*th*^ and *j*^*th*^ NP at distance *r* given by^40^

1 $$\frac{{{U}}_{{ij}}({r})}{{{k}}_{{B}}{T}}=\,\frac{{{z}}_{{i}}{{z}}_{{j}}{{\lambda }}_{{B}}{{\sigma }}_{{\rm{0}}}}{(1+\,\frac{{\kappa }{{\sigma }}_{{i}}}{2})(1+\,\frac{{\kappa }{{\sigma }}_{{j}}}{2})}\frac{{{e}}^{{\kappa }{{\sigma }}_{{ij}}}{{e}}^{-{\kappa }r}}{{r}}.$$

The soft-core repulsion between NP is modeled using Lennard-Jones (LJ) potential given by

2 $$\frac{{{U}}_{{ij}}^{{LJ}}({r})}{{{k}}_{{B}}{T}}=4{{\varepsilon }}_{{ij}}^{{LJ}}[{(\frac{{{\sigma }}_{{ij}}}{{r}})}^{12}-\,{(\frac{{{\sigma }}_{{ij}}}{{r}})}^{6}]$$

where *i*, *j* = *A*, *B* and *σ*_*ij*_ = (*σ*_*i*_ + *σ*_*j*_)/2 is the average diameter of *i*^*th*^ and *j*^*th*^ NPs. We define dimensionless Bjerrum length as ${\lambda }_{B}=\,{e}^{2}/4\pi \varepsilon {\varepsilon }_{0}{k}_{B}T{\sigma }_{0}$ and Debye screening length as ${\kappa }^{-1}={(8\pi {\lambda }_{B}{\sigma }_{0}{c}_{s})}^{-\frac{1}{2}}$, where *σ*_0_ = (*σ*_*A*_ + *σ*_*B*_)/2. *ε*, *ε*_0_, *k*_*B*_, T and *c*_*s*_ are the dielectric constant of solvent, permittivity in free space, Boltzmann constant, absolute temperature and salt concentration respectively. In simulation we have used truncated and shifted form of both electrostatic and soft-core potential to improve computational efficiency, that is,

3 $$\frac{{{U}}_{{ij}}^{{c}}({r})}{{{k}}_{{B}}{T}}=\{\begin{array}{c}{{U}}_{{ij}}({r})-{{U}}_{{ij}}({{r}}_{{ij},{cut}})\,\\ 0\end{array}\,\begin{array}{c}{r}\le {{r}}_{{ij},{cut}}\\ {r} > {{r}}_{{\boldsymbol{i}}{j},{cut}}\end{array}$$

and

4 $$\frac{{{U}}_{{ij}}^{{LJ},{c}}({r})}{{{k}}_{{B}}{T}}=\{\begin{array}{c}{{U}}_{{ij}}^{{LJ}}({r})-{{U}}_{{ij}}^{{LJ}}({{r}}_{{ij},{cut}}^{{LJ}})\\ 0\end{array}\,\begin{array}{c}{r}\,\le \,{{r}}_{{ij},{cut}}^{{LJ}}\\ {r} > {{r}}_{{ij},{cut}}^{{LJ}}\end{array}$$

Here *r*_*ij*,*cut*_ = 2.5*σ*_*ij*_ and ${r}_{ij,cut}^{LJ}={2}^{1/6}{\sigma }_{ij}$ are cutoff distances for Columbic interaction and softcore LJ repulsion respectively. We have not included the Van der Walls interactions in our simulations because we are interested in studying the effect of electrostatic interactions on aggregation kinetics and strength of electrostatic interactions we are using are of the order of 80k_B_T.

Simulation algorithm comprises of following steps^38^:

- Random distribution of NPs inside cubic box of length $L=\,{[\frac{\pi }{6\eta }({\sigma }_{A}^{3}{n}_{A}+{\sigma }_{B}^{3}{n}_{B})]}^{1/3}$, where *η* is the packing fraction, defined as the fraction of volume occupied by NPs.
- Trial displacement of the center of a randomly picked NP to the surface of a sphere of radius *a* around the center of NP. We choose a random point ($\theta =2\pi u,\phi ={\cos }^{-1}(2v-1)$) on the surface of sphere where *u* and *v* are uniformly distributed random variables between (0, 1); *θ* and *φ* are the polar angle and azimuthal angle, respectively.
- Move is accepted or rejected using the Glauber transition probability $p=\,{[1+{e}^{{\rm{\Delta }}E/{k}_{B}T}]}^{-1}$, where Δ*E* is the energy change for the trial displacement.

One kMC sweep consist of *N*_*T*_ trial moves. Time step Δ*t* of a sweep is related to the step size *a* as ${\rm{\Delta }}t=\frac{{a}^{2}}{12D}$, where D is the diffusion coefficient of NP. For NP of diameter *σ*_0_, characteristic diffusion time scale is ${\tau }_{0}=\frac{{\sigma }_{0}^{2}}{D}\Rightarrow {\rm{\Delta }}t=({\tau }_{0}/12){(a/{\sigma }_{0})}^{2}$. In theory, Δ*t* is calculated by assuming the energy change in each sweep is small. In previous kMC study^39^, *a* = 0.02*σ*_0_ is observed as the proper step size and we have used this value of *a* in our simulation. Note that the current approach do not explicitly account for changes in diffusion coefficient on aggregation, as elaborated in previous studies^36,38,39^. However, this limitation is not expected to be of much consequence in the current work, since we focus on early stage kinetics of NP aggregation when the aggregate size is relatively small except for the high particle concentration case where percolation occurs.

### Experiment (Materials and Method)

Chloroform, SDS, CTAB, PMAA and acetone were purchased from Sigma–Aldrich. All the materials were used as obtained. Millipore water was used in all parts of experiment including NP synthesis and DLS study. We synthesized both positively and negatively charged PMAA NPs of different concentrations with mini-emulsion technique using anionic surfactant, SDS (sodium dodecyl sulfate) and cationic surfactant, CTAB (cetyltrimethyl ammonium bromide), respectively. In a typical fabrication process, SDS/CTAB and PMAA are separately dissolved in distilled water and chloroform, respectively. Polymer solution was heated to 50 °C to ensure complete solubility. The NPs were then formed by adding the polymer solution to the aqueous SDS/CTAB solution under probe sonication with constant heat environment at 50 °C and stirring at 1000 rpm by hot plate. After complete cycle of probe sonication for 6 minute, dispersion was kept on heating at 65 °C for 45 minute to remove chloroform. To perform aggregation kinetics experiment, we prepared two sets by mixing both positively and negatively charged NPs together. In set 1, 200 ml was prepared by mixing distilled water with 25 *μl* of each positive and negative NPs. Similarly in set 2, 50 *μl* of each positive and negative NPs were mixed in distilled water to get 200 ml solution. Average aggregate size(*M*_*N*_) and zeta potential(ζ) of NPs was recorded by Malvern Nanosizer DLS instrument.

## Results and Discussion

### Simulation Results

We numerically studied the aggregation kinetics for the following three sets of oppositely charged NPs:

1. *Symmetric NPs*: Both type A and type B have same size (*σ*_*A*_ = *σ*_*B*_) and equal and opposite valence (*z*_*A*_ = −*z*_*B*_).
2. *Charge Asymmetric NPs*: Both type A and type B have same size (*σ*_*A*_ = *σ*_*B*_) but unequal valence (*z*_*A*_ = −2.0*z*_*B*_).
3. *Size Asymmetric NPs:* type A and type B have unequal size (*σ*_*A*_ = 1.5*σ*_*B*_) but equal and opposite valence (*z*_*A*_ = −*z*_*B*_).

Starting from random distribution of NPs inside the simulation box we computed the aggregate size distribution of NPs at different times for three different packing fractions (*η* = 0.005,0.025,0.06). Aggregate size of a cluster is calculated by computing center to center distance(*r*) between two NPs and two NPs are considered to be part of the same cluster if *r* < 1.5σ_0_. We computed mean average aggregate size (${M}_{N}=\sum _{j}{n}_{j}j/\sum _{j}{n}_{j}$), weight average aggregate size (${M}_{w}=\sum _{j}{n}_{j}{j}^{2}/{n}_{j}j$) and mean radius of gyration square $({R}_{g}^{2}=\sum _{i=1}^{{N}_{c}}{R}_{g,i}^{2}/{N}_{c})$ as a function of kMC steps, where the number of aggregates of size *j* is *n*_*j*_, *N*_*c*_ ($={\sum }_{j}{n}_{j}$) is total number of aggregates in the system. ${R}_{g,i}^{2}=\frac{1}{{N}_{i}}\sum _{j=1}^{{N}_{i}}{({{\boldsymbol{r}}}_{{\boldsymbol{j}}}-{{\boldsymbol{r}}}_{{\bf{0}}})}^{2}$ is the radius of gyration square of i-th aggregate of size *N*_*i*_ and center of mass ${{\boldsymbol{r}}}_{0}=\frac{1}{{N}_{i}}\sum _{j=1}^{{N}_{i}}{{\boldsymbol{r}}}_{{\boldsymbol{j}}}$. We have chosen simulation parameters, λ_B_ = 81, κ^−1^ = σ_0_ such that we are working in the NPs aggregation regime^36^.

**Figure 1:**
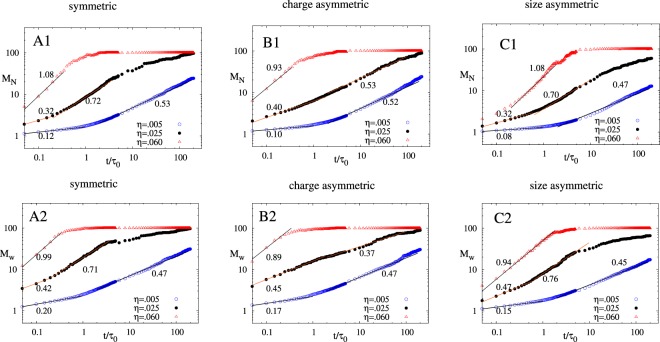
A1(A2), B1(B2), C1(C2) show *M*_*N*_(*M*_*w*_) *vs t*/*τ*_0_ plot for symmetric, charge asymmetric and size asymmetric case for three different values of η respectively. Solid lines indicate the power law fit for different time windows to the simulation data (η = 0.005 ($\odot$), 0.025(●), 0.06(Δ)) averaged over 50 different simulations starting with different initial configuration. Power law exponent to the fit are shown in the plot. Parameters for the simulation for all three cases are ${{N}}_{{T}}=100,{{\lambda }}_{{B}}=81,{{\kappa }}^{-1}={{\sigma }}_{0},{{\varepsilon }}_{{ij}}^{{LJ}}=1$.

Figure 1 shows the time evolution of *M*_*N*_ and *M*_*w*_ of all the three sets for three different packing fractions (η = 0.005 ($\odot$), 0.025(●), 0.06(Δ)). We observed two different aggregation kinetics regimes and time dependence of average aggregate size can be written as ${M}_{N}\sim {t}^{{\beta }_{1}}$ (initial phase) and ${M}_{N}\sim {t}^{{\beta }_{2}}$ (intermediate phase) for all three sets. The *β*_1_ and *β*_2_ values characterize aggregation rate at short and intermediate time respectively. Both *β*_1_ and *β*_2_ strongly depend on the concentration of the NPs in the system as evident from Fig. 1. For *η* = 0.06, *β*_1_ (short time aggregation regime) was not captured due to initial fast clustering and only *β*_2_ values are given in Fig. 1. However, initial kinetics regime is slightly visible for *η* = 0.06 in size asymmetric case where aggregation is slow due to large size of the NPs. As η increases, both *β*_1_ and *β*_2_ increases for all the three cases. But, there is no substantial difference in *β*_2_ values for *η* = 0.025 and *η* = 0.005 in charge asymmetric case. Furthermore, for all the three sets, we observed *β*_2_ > *β*_1_ for all η, but again in charge asymmetric case this difference is not very predominant as *η* increases. The behavior of exponents *β*_1_ and *β*_2_ in charge asymmetric case for intermediate to high *η* hints towards anomalous behavior of charge asymmetric system. We further plotted *M*_*w*_ vs t/τ_0_ and observed similar time dependence behavior as *M*_*N*_ vs t/τ_0_, therefore we write ${M}_{w}\sim {t}^{{\beta }_{1}^{^{\prime} }}$ at initial phase and ${M}_{w}\sim {t}^{{\beta ^{\prime} }_{2}}$ at intermediate phase. The exponents ${\beta }_{1}^{^{\prime} }$ and ${\beta }_{2}^{^{\prime} }$ behaves very similar to *β*_1_ and *β*_2_ respectively, except for charge asymmetric case where ${\beta }_{2}^{^{\prime} }$ for *η* = 0.025 is smaller than ${\beta }_{1}^{^{\prime} }$ for *η* = 0.005. Also, ${\beta }_{2}^{^{\prime} } > {\beta }_{1}^{^{\prime} }$ is only valid for low values of η (see Fig. 1B2) for charge asymmetric case, where for $\eta =0.005,{\beta }_{2}^{^{\prime} } > {\beta }_{1}^{^{\prime} }$ but for $\eta =0.025,{\beta }_{2}^{^{\prime} } < {\beta }_{1}^{^{\prime} }$. The anomalous aggregation kinetic behavior for intermediate to high particle densities that was indicated by *M*_*N*_ exponents is distinctly visible from *M*_*w*_ exponents for the charge asymmetric case.

**Figure 2:**
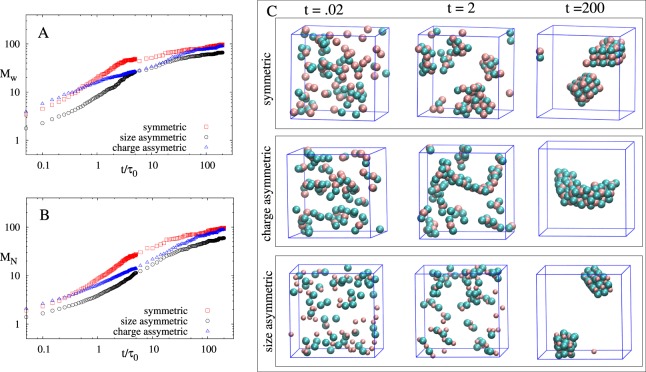
(**A**) shows *M*_*w*_ *vs t*/*τ*_0_ and (**B**) shows *M*_*N*_ *vs t*/*τ*_0_ for *η* = 0.025 for all three cases. Crossing of aggregate size is visible between symmetric and charge asymmetric case. (**C**) Shows the snapshots of NP aggregation for three different time (pink and cyan color represents positively and negatively charge NPs, respectively). Parameters for the simulation for all three cases are ${{N}}_{{T}}=100,{{\lambda }}_{{B}}=81,{{\kappa }}^{-1}={{\sigma }}_{0},{{\varepsilon }}_{{ij}}^{{LJ}}=1$.

**Figure 3:**
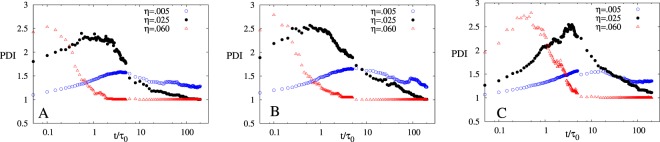
Polydispersity, *PDI vs t*/*τ*_0_ plot of (**A**) symmetric, (**B**) charge asymmetric and (**C**) size asymmetric for three different values of η (η = 0.005 ($\odot$), 0.025(●), 0.06(Δ)) averaged over 50 different simulations starting with different initial configuration. Parameters for the simulation for all three cases are ${{N}}_{{T}}=100,{{\lambda }}_{{B}}=81,{{\kappa }}^{-1}={{\sigma }}_{0},{{\varepsilon }}_{{\boldsymbol{i}}{j}}^{{LJ}}=1$.

The increase in the exponent *β*_1_ and ${\beta }_{1}^{^{\prime} }$ as *η* increases is due to decrease in the average separation between NPs. This results in an increase in the Columbic force between NPs and hence faster aggregation at initial time for large η values is observed. Strong Columbic attraction due to high charge on NPs leads to much faster initial aggregation in charge asymmetric case as compared to symmetric and size asymmetric case (see Fig. 2A,B, blue data points are above red for short time). The aggregation become even faster at intermediate time for all the cases except for larger η values in charge asymmetric case (see Fig. 1). At intermediate time there are two processes that will determine the growth of the aggregate - one is the strength of electrostatic interactions between the NP clusters present in the system that determine how fast the clusters agglomerate and another is the size of the clusters that are agglomerating. The interplay between these two processes will determine the *β*_2_ and ${\beta }_{2}^{^{\prime} }$ behavior. At initial time, small aggregates mainly comprising of dimers or trimer starts forming (see Fig. 2C, t = 0.02), rate of which depend on the concentration of particles. In charge symmetric systems dimers are more stable whereas in charge asymmetric case trimers are more favorable due to neutralization of charge on the cluster. These dimers and trimer formed in the system will now interact with other small clusters present in the system which are mostly dimers and trimers. Dimers will behave like electric dipoles and trimer will behave like electric quadrupole^41^. The interaction between

two dimers or trimers will depend on the electric field generated by these small clusters. For the sake of simplicity for a diluted solution we can assume that electric field $E\,\propto \,\frac{1}{{r}^{3}}$ for dimers and $E\,\propto \,\frac{1}{{r}^{4}}$ for trimers, where *r* is the distance of the point from the cluster where electric field is calculated. This shows that the interaction between dimers will be stronger than trimers and hence the agglomeration become slower in the case of asymmetric charge at intermediate time when compared to the symmetric case. We should emphasize here that the actual interaction will also depend on the orientation of clusters with respect to each other. Moreover, if the solution is not sufficiently dilute, other higher order terms like $\frac{1}{{r}^{5}},\frac{1}{{r}^{6}}$ etc. cannot be neglected from the electric field expression. In Fig. 2B (*t* = 0.02 snapshot), one NP of high valence (charge asymmetric case) is surrounded by many NPs of opposite charge which leads to more screening and excluded volume effect^42^ at intermediate time. This leads to large reduction in the strength of electrostatic interaction between the clusters and hence slower agglomeration of clusters. The increase in the size of agglomerated cluster due to the merging of smaller clusters is dominated by slower agglomeration of clusters in charge asymmetric case despite the fact that the asymmetric case have bigger clusters to agglomerate as compared to the symmteric case. Thus, we observe a crossing of aggregate size between symmteric and charge asymmetric case (see Fig. 2A,B). We further calculated polydispersity index (PDI) which is defined as the ratio of weight average aggregate size and number average aggregate size and given by *PDI* = *M*_*w*_/*M*_*N*_. Figure 3 show *PDI* vs t/τ_0_ plot for all the three cases for different packing fractions. PDI is low at initial time due to presence of single NPs and very small clusters, it increases as time increases and reaches a maximum value at intermediate times. As time further increases, small aggregates start coalescing to form large aggregates which again leads to a decrease in PDI. At large times, PDI value saturates at *PDI* = 1 (corresponding to monodispersed case) for large η values which represent the presence of a single large percolated cluster. Also, at initial time PDI value is higher for larger η which indicates that aggregation is faster for high η. This further substantiates our point that initial time aggregation behavior is not captured for higher η values. Another important point to notice is that the time corresponding to the maxima of PDI (see Fig. 3) is directly proportional to the crossover time between two scaling regimes observed in aggregate size dynamics (see Fig. 1). Simulation starts with the initial state of PDI = 1, i.e. homogenous system, but as the system evolves PDI increases (see Fig. 3), which implies that the heterogeneity in the system increases. At the maxima of PDI, we have very heterogeneous system (many different size clusters with different charges) and we can predict the aggregation kinetics of this heterogeneous system by looking at the evolution of the system from this point onward. Now, comparing Fig. 1 and Fig. 3 we can easily conclude that time corresponding to the maxima of PDI is always greater than the crossover time between the first and second scaling regimes. Therefore, for a highly heterogeneous system, we can conclude that aggregation kinetics will have only one scaling regime.

**Figure 4:**
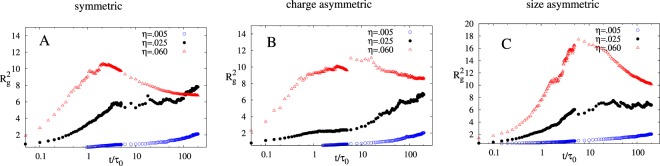
${{R}}_{{g}}^{2}\,{vs}\,{t}/{{\tau }}_{0}$ plot of (**A**) symmetric, (**B**) charge asymmetric and (**C**) size asymmetric for three different values of η (η  = 0.005 ($\odot$), 0.025(●), 0.06(Δ)) averaged over 50 different simulations starting with different initial configuration. Parameters for the simulation for all three cases are ${{N}}_{{T}}=100,{{\lambda }}_{{B}}=81,{{\kappa }}^{-1}={{\sigma }}_{0},{{\varepsilon }}_{{ij}}^{{LJ}}=1$.

Next, we plotted the radius of gyration square (${R}_{g}^{2}$) vs time in Fig. 4. Here we found an interesting non-monotonic behavior of ${R}_{g}^{2}$ for high value of *η* and plateau like behavior in case of intermediate *η*. The peak arises for higher values of η due to formation of a single big aggregate that spans the whole simulation box, but we observe formation of fibril like structure at initial to intermediate times (see Fig. 2C). The system further evolves to minimize free energy, and therefore whole cluster evolves to compact form giving rise to a decrease in the ${R}_{g}^{2}$. For intermediate and small η values, system evolves very slowly as compared to high η values hence it gets sufficient time to restructure its clusters and thus we get a saturation or plateau like structure instead of maxima. Another interesting point to notice is that the peak in case of charge asymmetric is much broader as compared to charge symmetric cases. This tells us that the rearrangement of fibril like structure to a more compact structure will start very slowly in charge asymmetric case. This might be due to the strong Columbic attraction between the NPs in charge asymmetric case as compared to symmetric and size asymmetric case. Henceforth, this leads to high barriers in the potential energy landscape of charge asymmetric system and thus makes NPs rearrangement difficult and therefore slow.

### Experimental Results

**Figure 5:**
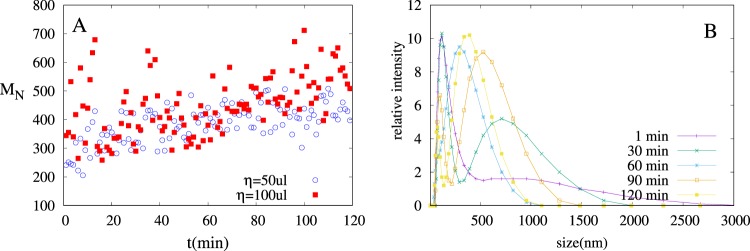
(**A**) Average aggregate size for two different concentration of oppositely charge NPs. *η* = 50 *μl* contain 25 *μl* of both positive and negative NPs in 200 ml solution and *η* = 100 *μl* contain 50 *μl* of both positive and negative NPs in 200 ml solution. (**B**) Particle size distribution at different times for *η* = 50 *μl*.

Positive NPs $$({M}_{N}=90\,nm,\zeta =47\,mV)$$ and negative NPs $$({M}_{N}=130\,nm,$$$$\zeta =-\,35\,mV)$$ are prepared using 10 *mg* CTAB and 5 *mg* SDS surfactant, respectively. Figure 5A shows time evolution of *R*_*H*_ of aggregates after mixing oppositely charged NPs for two different concentrations.

As we can observe in Fig. 5A, aggregation is dominant and increasing the particle concentration increases the aggregation rate. Fluctuations in aggregate size, as clearly visible in Fig. 5A, might indicate the non-homogeneity of the mixture. Figure 5B shows the relative intensity of particles of different size at a particular time. We observed that peaks are shifting towards right that indicates the increase in the average size of the aggregates. Furthermore, intensity profile is getting broader also with time, which indicates that poly-dispersity of the system is increasing with time. At different times we also observed bimodal nature in intensity profile for example in Fig. 5B, see time  = 30, 90 min. Bimodality confirms the non-homogeneity of the mixture. We did not observe two regimes in aggregation kinetics as observed in numerical simulation. One reason for this might be initial heterogeneity of the mixture, as explained earlier (i.e., starting from PDI maxima in simulations). Another possibility is that the NP used in the experiments are polymeric nanoparticles, which are coated with surfactant on the surface and they do not behave exactly like spherical NPs as we have assumed in numerical model. More controlled experiments are underway to study the system kinetics in more detail.

## Conclusion

In conclusion, we studied the effect of charge and size asymmetry of NPs on their aggregation kinetics and compared it with the symmetric system. We found two aggregation regimes in the aggregation kinetics of three different systems viz. symmetric, charge asymmetric and charge asymmetric. The aggregation kinetics is strongly dependent on the concentration of the NPs in the system. At initial time for a particular concentration, Columbic forces between NPs decides the aggregation rate and therefore charge asymmetric system containing NPs of high valence aggregates faster than symmetric systems (both size symmetric and size asymmetric). This also leads to large decrease in charge/size ratio of newly formed aggregates in charge asymmetric system as compared to symmetric system and hence at intermediate times, aggregation become slower in asymmetric system. This happens because of the interplay between the rate of agglomeration of clusters and the size of agglomerating clusters. Further, NPs within a cluster rearrange themselves to minimize free energy and due to interplay between the time scale of aggregation and time scale of rearrangement leads to a non-monotonic behavior in ${R}_{g}^{2}$. In charge asymmetric case rearrangement of NPs is difficult within a cluster due to presence of strong Columbic interactions between oppositely charged NPs and therefore, ${R}_{g}^{2}$ shows broader peak for charge asymmetric case as compared to symmetric case. We also performed few preliminary experiments on two sets of NPs and DLS studies confirm the dependence of particle concentration on aggregation kinetics. Due to organic group present on the nanoparticle surface, it is difficult to derive any power law from experiments and further experimental optimization is underway.
